# Interactions Between Morel Cultivation, Soil Microbes, and Mineral Nutrients: Impacts and Mechanisms

**DOI:** 10.3390/jof11060405

**Published:** 2025-05-24

**Authors:** Yiwen Fu, Muxin Fan, Haiyan Qin, Zeyu Zhang, Shijun Liu, Shuwen Wu, Yun Wang, Xia Yuan

**Affiliations:** 1 School of Life and Environmental Sciences, Hangzhou Normal University, Hangzhou 311121, China; fuyiwen@caas.cn (Y.F.); 13133392524@163.com (M.F.); qhy10427@163.com (H.Q.); zhangzeyu33@163.com (Z.Z.); 2022210314015@stu.hznu.edu.cn (S.L.); 18258292210@163.com (S.W.); 2024112010030@stu.hznu.edu.cn (Y.W.); 2 School of Environment and Surveying Engineering, Suzhou University, Suzhou 234000, China

**Keywords:** morels, soil microbes, soil mineral elements, soil physicochemical properties

## Abstract

Morel mushrooms (*Morchella* spp.) are prized for their nutritional and medicinal value. Despite extensive research on their cultivation, the species’ impacts on the soil microbiota and minerals remain unclear. This study systematically evaluated six *Morchella* species, analyzing their effects on soil physicochemical properties, microbial communities, and mineral nutrients levels. The results showed that yield varied significantly among the species, with an order of *M. sextelata* > *M. exuberans* > *M. eximia* > *M. importuna* > *Mel-13* > *Mel-21*. Cultivation led to a consistent reduction in soil NO_3_^−^-N levels, particularly in *M. eximia* and *Mel-13* (40–50% decrease), while NH_4_^+^-N levels did not change significantly, indicating mycelial nitrate preference. Mineral nutrient alterations exhibited distinct species-dependent patterns, with *M. eximia* showing the greatest increase in exchangeable Ca, while *M. importuna* and *M. sextelata* caused the most obvious decreases in available B. *Morchella* species exerted more pronounced impacts on fungal than bacterial communities, evidenced by significant reductions in alpha diversity—particularly in high-yield *M. sextelata*—suggesting species-specific fungal inhibition. At the phylum level, consistent depletion of *Ascomycota* but enrichment of *Mortierellomycota* were noted. Moreover, correlation analyses identified significant positive associations between morel yield and both fungal community diversity and exchangeable Ca content. This implies that *Morchella* species restructures soil fungal communities through nutrient competition and mineral-mediated regulation, with calcium acting as a key modulator. Overall, by elucidating the interconnected ‘*Morchella* species–microbe–mineral’ relationships, this study highlights *Morchella* species’ distinct regulation of soil microecology, providing valuable insights for the selection of optimized species like *M. eximia* and targeted soil management in morel cultivation.

## 1. Introduction

Morels (*Morchella* spp.), belonging to the *Ascomycota* phylum, are highly valued prized edible fungi renowned for their distinctive flavor and multifaceted nutritional, health, and medicinal benefits. As such, they have become highly sought after in the international market [1,2]. Nonetheless, their naturally short fruiting season and challenging growing conditions severely limit wild morel production, making them scarce; as such, they fail to meet the market demand. Even in controlled cultivation environments, their growth is inevitably influenced by various factors, such as physicochemical parameters and interactions with other biotic components [3,4]. However, the underlying mechanisms governing the interactions between different *Morchella* species and these influencing factors are still not fully understood and require in-depth investigation.

In recent years, various cultivation techniques and diverse species have been investigated to increase biomass yields [3,5]. The main species used have been *M. importuna*, *M. sextelata*, and *M. eximia* of the black morel mushroom, with outdoor cultivation of morels becoming a large-scale commercial practice in China. However, the cultivation process of morels is full of instability due to the intricate interplay between their mycelial growth, fruiting body formation, and soil physicochemical factors such as pH, carbon source type, soil mineral content, salinity, and temperature [6,7]. Additionally, morel yields can also be reduced by successive cultivation in field experiments [8]. Specifically, morel growth can deplete the available phosphorus in the soil while increasing total potassium levels and soil pH. It also increases organic matter (e.g., effective nitrogen) and the activity of enzymes like sucrase and amylase [9]. One hypothesis suggests that this may occur because the growth of morels absorbs necessary nutrients from the soil while the metabolism of certain substances in the soil accumulates, potentially leading to nutrient imbalances and changes in other physicochemical properties, which, in turn, affect the soil microbial community [7]. Furthermore, morels significantly enrich some metal elements in the soil, effectively improving soil fertility and offering great prospects for soil remediation. Various mineral elements significantly affect mycelial growth and soil microbial structure [7,10,11]. Notably, the elemental content of iron (Fe), zinc (Zn), manganese (Mn), and copper (Cu) has a profound effect on the growth and ascospore formation of morels. For example, research has shown that Mn supplementation significantly increases mycelial growth rates, resulting in higher yields [12].

Moreover, soil microbial communities play a key role throughout the morel growth cycle [13]. Studies have highlighted their influence on morels, often even exceeding the influence of soil physicochemical properties [14]. For instance, a multi-site study showed that the high production of morels was primarily due to the high alpha diversity of the soil fungal community, rather than the soil physiochemical characteristics. This highlights the importance of microbial ecology in the large-scale agroindustry of soil-cultivated mushrooms [15]. Further research has also explored the relationships between the adaptation of *Morchella* spp. and specific microbial functional groups, showing a shift from photoautotrophic N-fixing bacteria on bare soil to pioneer heterotrophs (such as oligotrophs and nitrifiers) in young *M*. *rufobrunnea*, then to saprobes, organic-N degraders, and other specialists under mature fruiting [16]. This suggests an influence of *M*. *rufobrunnea* on the soil prokaryotic microbiome and nutrition. In addition, specific bacterial groups, such as *Pedobacter*, *Pseudomonas*, *Stenotrophomonas*, and *Flavobacterium*, which are commonly abundant in morel substrates, form the core microbiome necessary for the development of *M*. *sextelata* ascocarps [17]. Yet, further research is required to explore changes in soil bacterial and fungal communities in response to diverse *Morchella* cultivation.

In this study, we comprehensively investigated the effects of morel cultivation on soil physicochemical properties, microbial communities, and mineral levels, elucidating their interactions and underlying regulatory mechanisms. Six predominant species currently cultivated in the Chinese *Morchella* industry for outdoor monoculture were selected, including *Morchella importuna*, *Morchella sextelata*, *Morchella exuberans*, *Morchella eximia*, *Morchella Mel-13* (*Mel-13*), and *Morchella Mel-21* (*Mel-21*). In-depth analyses of the post-harvest morel yield and the corresponding soil samples were carried out separately, evaluating different soil physico-chemical indicators, mineral element levels, and microbiological indicators (bacterial and fungal community structure, and enzyme activity). The objectives included the following: (1) to determine the species-specific impacts of *Morchella* cultivation on soil mineral element levels; (2) to characterize the effects of morel cultivation on soil bacterial and fungal community diversity, composition, and functional potential; and (3) to elucidate the correlations among soil microbial communities, mineral element availability, and morel productivity.

## 2. Materials and Methods

### 2.1. Experimental Design

**Figure 1:**
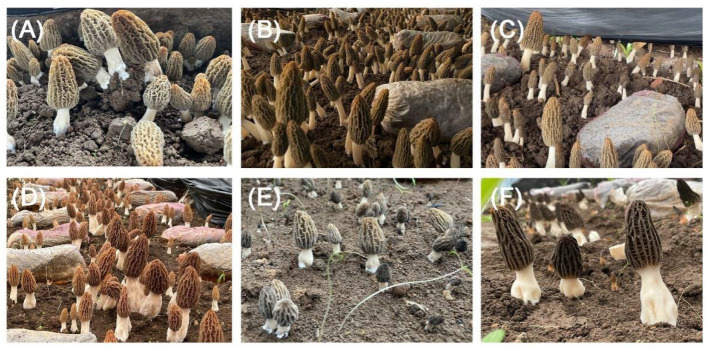
Six Morchella species (**A**–**F**). (**A**) *M. importuna*; (**B**) *M. sextelata*; (**C**) *M. exuberans*; (**D**) *M. eximia*; (**E**) *Mel-13*; (**F**) *Mel-21*.

This study focused on six major *Morchella* species widely cultivated in China: *M. importuna*, *M. sextelata*, *M. exuberans*, *M. eximia*, and *Mel-13* and *Mel-21* (Figure 1A–F). The experimental site comprised an approximately one-hectare experimental area (100 m × 100 m) located at the Anhui Agricultural University experiment station in Suzhou city, Anhui province (33.69° N, 117.09° E). Within this area, seven greenhouses, each measuring 30 m × 10 m and covered with 80% shading nets, were randomly positioned with a minimum distance of 10 m between them to reduce bias. Six greenhouses were used for cultivating the different *Morchella* species, while one served as a control without morel cultivation. Prior to sowing, the soil was tilled to a depth of 15–20 cm using a rotary tiller to ensure uniformity.

In December 2023, spawn and cultivation medium (78% wheat, 20% corn cob, 1% lime, and 1% gypsum) were sown in each greenhouse using a seed drill. The mixture was uniformly distributed at 0.3 kg m^−2^ and a consistent depth, enhancing efficiency and distribution over manual methods. Then, nutrient bags (1 kg, 50% wheat + 48% corn cob + 1% lime + 1% gypsum) were placed 7–10 days post-sowing at a density of 3 bags m^−2^. Black PE film with holes was covered to keep the soil moisture content above 20%, and the CO_2_ content of the greenhouses was kept below 2000 ppm by adjusting the ventilation. At the fruiting stage, water was sprayed again to penetrate the soil to a depth of 20 cm. An arched shed covered with white PE film with small holes was established at a height of 30 cm above the ground for moisturizing. And in the arched shed, the air temperature was below 20 °C, the air humidity was above 85%, and the CO_2_ content was below 2000 ppm.

The fruiting bodies were harvested in March and April in 2024. The average yield, measured in fresh weight, was calculated by dividing the total weight of the fruiting bodies from each greenhouse by the cultivation area. Post-harvest, three 2 m × 2 m sampling plots were established in each greenhouse, positioned at least 4 m apart from each other and more than 2 m from the greenhouse edges. Surface soil samples from a 0 to 10 cm depth were collected from each plot using the five-point sampling method and pooled to create a single composite sample. A total of 21 soil samples were collected (7 treatments including the control, with 3 replicates each). These samples were extracted with a soil auger, placed in a cooler box, and promptly returned to the laboratory. After removing debris such as stones and other impurities in the lab, each soil sample was divided into three portions. One portion was air-dried for the determination of basic physicochemical indicators, including pH value. Another portion was stored in a refrigerator for the assessment of enzyme activity and available nutrient content within 3 days. The third portion was stored at −80 °C for the extraction of soil microbial DNA for microbial amplicon sequencing.

### 2.2. Soil Physicochemical Properties

The soil moisture content was determined by the drying method, with drying performed at 105 °C to a constant weight. Soil electrical conductivity (EC) was measured using the electrode method on a mixture of soil and deionized water at a ratio of 1:1.5. Soil pH was assessed with a pH meter (Thermo Orion-868, Thermo, San Jose, CA, USA) in a mixture of soil and deionized water (soil/water, 1:2.5) [18]. After removing the inorganic carbonate with 1 M HCl, soil organic carbon (SOC) and total nitrogen (TN) were quantified by an elemental analyzer [19]. The available nitrogen (AN) in the soil, including NH_4_^+^-N and NO_3_^−^-N, was measured by the UV spectrophotometric method for NO_3_^−^-N and the indophenol blue colorimetric method for NH_4_^+^-N. Available phosphorus (AP) was determined using Olsen’s method [20]. The available metals in the soil, including the available zinc (AZn), manganese (AMn), cuprum (ACu), and iron (AFe), were extracted by diethylenetriaminepentaacetic acid (DTPA) and analyzed by atomic absorption spectrophotometry. The available boron (AB) in the soil was extracted with boiling water, the interference of iron and aluminum ions was eliminated by EDTA, the color of organic matter was removed by potassium permangate, and the amount of boron in the extracted solution was determined by methylenimine -H colorimetric method. Soil available potassium (AK) and exchangeable sodium (ENa) was extracted with an ammonium acetate solution and determined by a flame photometer. Soil exchangeable calcium (ECa) and magnesium (EMg) were extracted by an ammonium acetate solution and determined by atomic absorption spectrophotometry.

### 2.3. Soil Enzymatic Activity

The activity of three hydrolases was detected by a microplate fluorimetric assay [21], including β–1,4–glucosidase (BG) involved in C–acquisition, β–1,4–N–acetylglucosaminnidase involved in N–acquisition (NAG), and acid phosphatase involved in P-acquisition (AP). Briefly, 1.5 g of fresh soil samples were weighed and the soil samples suspended in 125 mL of Tris–HCl (pH = 7.5) buffer. The relevant reagents and soil slurries were added to the corresponding wells of a 96-well black microplate and incubated at 25 °C for 3 h. Then, the fluorescence was measured at an excitation wavelength of 360 nm and an emission wavelength of 450 nm using a Fluorescence Microplate Reader (SynergyH1M, BioTek, Winooski, VT, USA). The units for hydrolytic enzyme activity were nmol g^−1^ dry soil h^−1^.

### 2.4. High-Throughput Sequencing of Microbial Communities

Microbial DNA was extracted from soil samples using the ALFA–SEQ kit (mCHIP Biotechnology Co., Ltd., Guangzhou, China). DNA concentration was measured by agarose gel electrophoresis. The diversity and taxonomic composition of the soil bacterial and fungal communities were characterized by high-throughput sequencing.

The 16S rRNA region of bacteria was amplified by PCR using the primers 515F/806R. The conditions were as follows: 5 min at 94 °C, followed by 32 cycles of denaturation at 94 °C for 30 s, annealing at 53°C for 30 s, and extension at 72 °C for 30 s, and finally, extension at 72 °C for 8 min. The fungal internal transcribed spacer (ITS1) gene was amplified using the primers BD–ITSIF/ITS2R. The PCR program consisted of initial denaturation at 95 °C for 3 min, followed by 34 cycles of denaturation at 95 °C for 20 s, annealing at 56 °C for 20 s, and extension at 72 °C for 30 s, followed by a final extension at 72 °C for 5 min. Finally, the DNA was sequenced on an Illumina MiSeq platform (Illumina, San Diego, CA, USA) to obtain the sequence list. The ASVs were then identified taxonomically using the Quantitative Insights Into Microbial Ecology 2 (QIIME 2) feature classifier plug-in. Taxonomic information was annotated in comparison with the SILVA (16S rRNA) and UNITE (ITS) databases.

### 2.5. Statistical Analysis

Statistical analyses were conducted using R (R Development Core Team 2022, R version 4.2.2). The impact of different morels on soil physicochemical properties, enzyme activity, and microbial community diversity and taxa were analyzed by a One-way ANOVA after tests of normality (Shapiro-Wilk test) and homogeneity of variance (Bartlett test). The post hoc comparison was performed using Tukey’s HSD test. The beta-diversity of bacterial and fungal communities was evaluated through principal coordinate analysis (PCoA) based on Bray-Curtis distances. The significance of the effect of different morels on community dissimilarity was tested using PERMANOVA with the adonis function in the “vegan” package. Pearson’s correlation coefficient analysis was conducted to analyze interactions between soil enzyme activity, soil physiochemical characteristics, bacterial and fungal OTUs, and changes in SOC and its molecular groups. Figures were plotted using R and GraphPad Prism 8.0.2.

## 3. Results

### 3.1. Yields of Different Morchella Species

**Figure 2:**
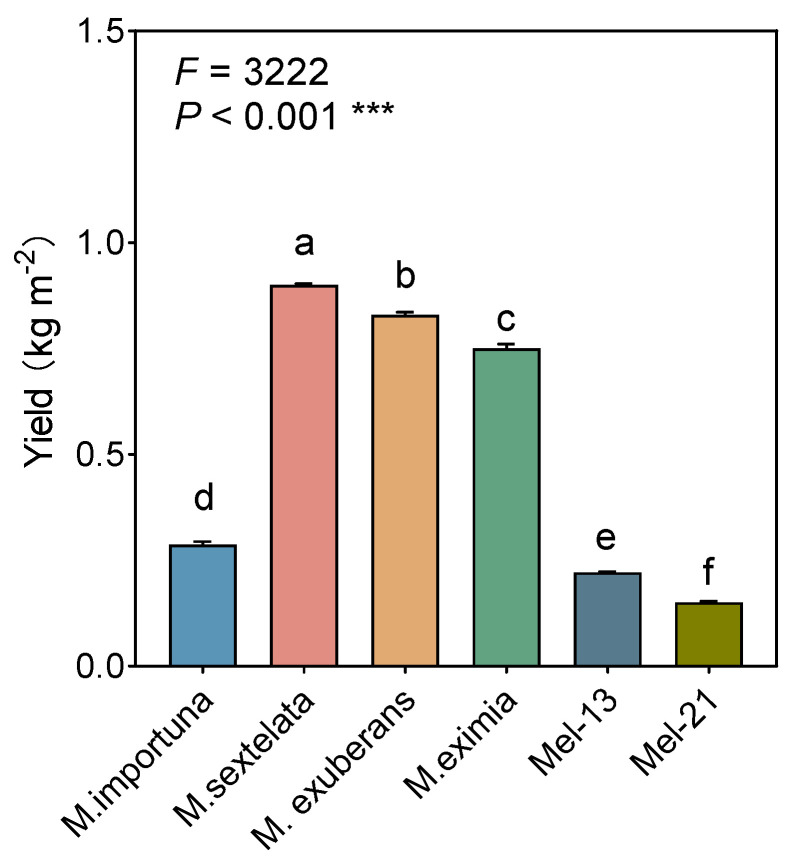
The yields of different *Morchella* species. Each value and bar are the repeated mean values and ± SE (*n* = 3). Significant differences between different morel treatments are indicated by lowercase letters (*** *p* < 0.001).

The annual yields of various *Morchella* species ranged from 0.15 to 0.90 kg m^−2^ (fresh weight), with significant differences observed among them. Notably, *M. sextelata* exhibited the highest yield, followed closely by *M. exuberans* and *M. eximia*. The yields of these three species were markedly superior, exceeding the yields of the other three types by more than double. Conversely, the yield of *Mel-21* was lowest among these six species, with a yield of 0.15 kg m^−2^ (Figure 2).

### 3.2. Soil Physiochemical Characteristics

**Figure 3:**
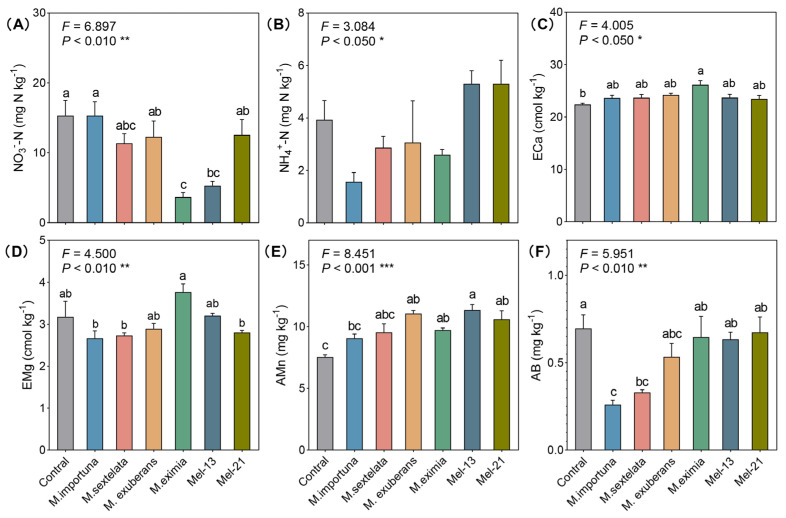
Soil physiochemical characteristics of cultivated *Morchella* species. (**A**) NO_3_^−^-N, nitrate N; (**B**) NH_4_^+^-N, ammonium N; (**C**) ECa, exchangeable Ca; (**D**) EMg, exchangeable Mg; (**E**) AMn, available Mn; (**F**) AB, available B. Each value and bar are repeated mean values and ± SE (*n* = 3). Significant differences between different morel treatments are indicated by lowercase letters (a–c; * *p* < 0.05, ** *p* < 0.01, *** *p* < 0.001).

The physiochemical properties of the soils exhibited limited but species-specific changes following morel cultivation (Table 1 and Figure 3). Among the six species tested in this study, only *M. eximia* and *Mel-13* caused a significant reduction in soil NO_3_^−^-N levels (*p* < 0.01, Figure 3A), while NH_4_^+^-N remained unchanged (Figure 3B). Under *M. eximia* cultivation, the levels of exchangeable Ca (ECa) and Mg peaked among all treatments, with ECa showing a significant increase compared to the control (Figure 3C), and EMg being significantly higher than in the *M. importuna*, *M. sextelata*, and *Mel-21* treatments (Figure 3D).

Consistent across all species, morel cultivation demonstrated a positive effect on increasing soil manganese (Mn) levels, particularly under *M. exuberans*, *M. eximia*, *Mel-13,* and *Mel-21* (Figure 3E). Conversely, AB levels were higher in the control group compared to morel cultivation, particularly under the *M. importuna* and *M. sextelata* treatments (Figure 3F). No statistically significant differences were observed in pH, SOC, or most other mineral elements (Table 1), suggesting that *Morchella* species selectively alter specific soil nutrients rather than inducing broad physicochemical shifts.

### 3.3. Enzyme Activity of Microorganisms

In general, morel cultivation had minimal discernible effects on soil hydrolase activity (Figure S1). Specifically, it exhibited a marginal positive influence on BG activity (*p* = 0.066, Figure S1A), whereas no significant impact was observed on NAG and AP activity (Figure S1B,C). Furthermore, although no statistically significant effects were detected, the values of BG, NAG, and AP in soils cultivated with morels were all higher than those in the control group.

### 3.4. Soil Bacterial and Fungal Community Structure

**Figure 4:**
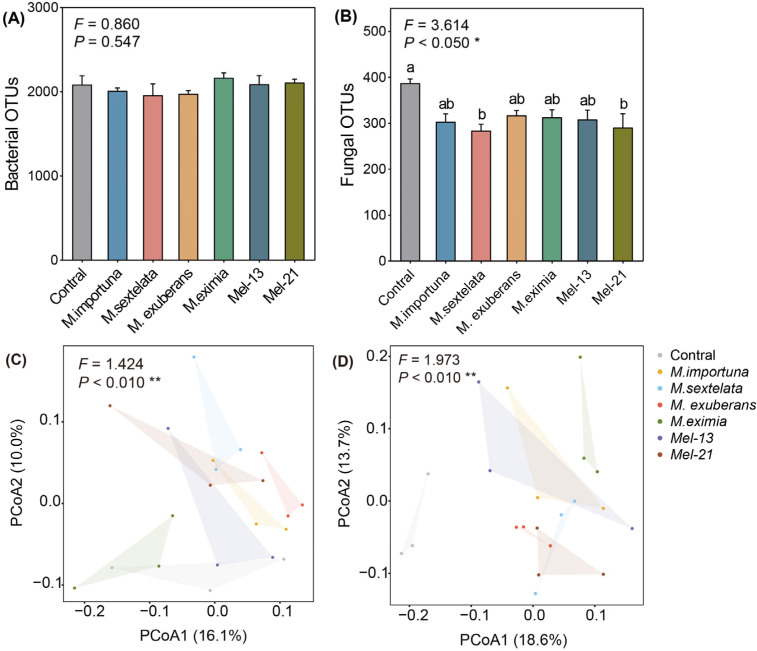
α and β diversity of soil bacterial and fungal communities of cultivated *Morchella* species. (**A**) bacterial OTUs; (**B**) fungal OTUs (each value and bar are repeated mean values and ±SE, *n* = 3). β diversity of bacterial (**C**) and fungal (**D**) communities were analyzed using PCoA based on Bray-Curtis distance metric. Significant differences between different morel treatments are indicated by lowercase letters (* *p* < 0.05, ** *p* < 0.01). Unmarked comparisons are not statistically significantly different (*p* > 0.05).

Microbial community responses were more pronounced in fungi than bacteria (Figure 4A,B). Morel cultivation influenced the richness but had no significant effect on the diversity of fungal communities, as indicated by the OTUs (operational taxonomic units) (Figure 4B) and Shannon diversity index (*H*’) (Figure S2). Moreover, PCoA based on the Bray-Curtis distance revealed distinct compositional differences in both bacterial and fungal communities (Figure 4C,D).

**Figure 5:**
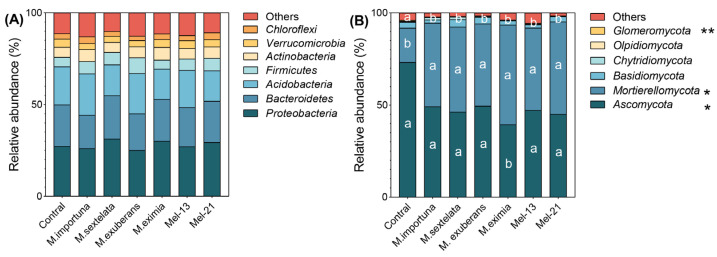
Relative abundance of dominant bacterial and fungal phyla in soil samples. (**A**) bacterial phyla; (**B**) fungal phyla. Significant differences between different morel treatments are indicated by lowercase letters (* *p* < 0.05, ** *p* < 0.01). Unmarked comparisons are not statistically significantly different (*p* > 0.05).

The dominant bacterial phyla across all soil samples were *Proteobacteria*, *Bacteroidete*, *Acidobacteria*, *Firmicute*, and *Actinobacteria* (Figure 5A). The relative abundance of each phylum was not significantly different between the treatment groups and CK. This reflects that morel cultivation did not significantly affect the relative abundance of dominant bacterial phyla.

*Ascomycota*, *Mortierellomycota*, *Basidiomycota*, *Chytridiomycota*, and *Olpidiomycota* were identified as the most dominant fungal phyla in all soil samples (Figure 5B). Compared with the soil fungal community of the CK group, the relative abundance of *Ascomycota* and *Olpidiomycota* was significantly lower, while that of *Mortierellomycota* was significantly higher. Notably, compared with the other treatment groups, the relative abundance of *Ascomycota* was the lowest and that of *Mortierellomycota* was the highest in the *M. eximia* treatment group. No statistically significant differences were observed in the relative abundance of *Basidiomycota*, *Chytridiomycota*, and *Glomeromycota*. This reflects that morel cultivation drives the significant phylum-level restructuring of soil fungal communities, with the most pronounced shifts occurring under *M. eximia* treatment.

### 3.5. Correlations Among Soil Microbial Communities, Mineral Element Availability, and Morel Productivity

**Figure 6:**
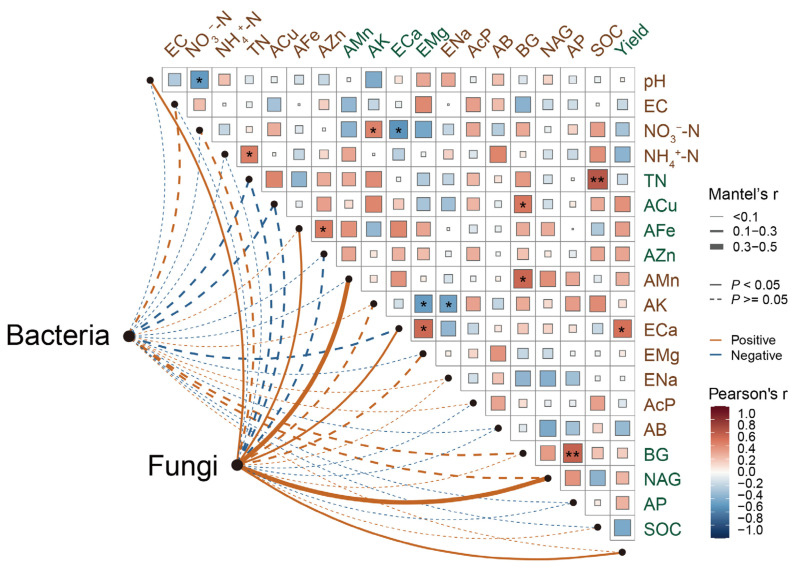
The correlations among soil physiochemical characteristics, including mineral element availability, bacterial and fungal OTUs, and morel productivity. The edge width indicates Mantel’s r statistic with the corresponding width. The color gradient indicates Pearson’s r correlation coefficient. The relation between bacterial and fungal OTUs and all variables was analyzed by a Mantel test. EC, electric conductivity; NO_3_^−^−N, nitrate N; NH_4_^+^−N, ammonium N; TN, total nitrogen; ACu, available Cu; AFe, available Fe; AZn, available Zn; AMn, available Mn; AK, available K; ECa, exchangeable Ca; EMg, exchangeable Mg; ENa, exchangeable Na; AcP, available P; AB, available B; BG, β–1,4–glucosidase; NAG, 1,4–N–acetylglucosaminidase; AP, acid phosphatase; SOC, soil organic carbon. Significance levels: * *p* < 0.05, ** *p* < 0.01.

According to correlation analyses and Mantel tests (Figure 6), fungal OTUs exhibited stronger correlations with soil enzyme activity compared to bacterial OTUs. However, soil bacteria did not show significant correlations with any specific soil varieties. Fungal OTUs were significantly (*p* < 0.05) and positively correlated with soil pH, AFe, AMn, ECa, and NAG and morel yield. Additionally, morel yield was positively correlated with ECa. NO_3_^−^-N had prominent negative correlations with pH and ECa while displaying a significant positive correlation with AK.

The correlations of *Ascomycota* and *Mortierellomycota* with key soil physicochemical characteristics and nitrogen-fixing bacteria showed opposite trends (Figure S3). *Mortierellomycota* demonstrated positive correlations with AFe, AMn, and ECa, as well as the bacterial genera *Bradyrhizobium* and *Pseudomonas*. In contrast, *Ascomycota* displayed negative correlations with these same factors. Moreover, *Ascomycota* had a positive association with NO_3_^−^-N and the genus *Burkholderia*, correlations that were inversely observed for *Mortierellomycota*.

## 4. Discussion

### 4.1. Effects of Morel Cultivation on Soil Microbial Communities

Morel cultivation exerts significantly negative impacts on the diversity of soil fungal communities. This finding is consistent with previous studies that reported a decline in fungal α diversity in morel-cultivated soils compared to uncultivated control soils [22,23]. This decline can be attributed to two primarily factors. First, the dominance of morels in the soil ecosystem may suppress the growth of other fungal taxa [23,24]. As a macrofungus, *Morchella* expands its mycelium, competing with other fungi for resources such as carbon and nitrogen. This competition inhibits the growth of other fungi, thereby altering fungal community composition and diversity. Given that fungi in soil play crucial roles in decomposing complex organic matter (e.g., lignin, cellulose) and forming symbiotic relationships with plants (e.g., mycorrhizae), such shifts in fungal community diversity may further influence soil health and ecosystem functioning. Second, continuous morel cultivation progressively altered soil nutrient profiles and also mineral elements like ECa, which exerted indirect but obvious effects on fungi communities. These findings align with the previous studies demonstrating calcium-mediated fungal community shifts [25].

The fungal communities in both morel-cultivated and uncultivated soils were predominantly composed of *Ascomycota*, *Mortierellomycota*, and *Basidiomycota*. Interestingly, fungal phyla exhibited distinct responses to morel cultivation. Specifically, the relative abundance of *Ascomycota* decreased, while that of *Mortierellomycota* increased. This divergent response may be attributed to multiple interacting factors. On the one hand, *Morchella* mycelia preferentially take up nitrate nitrogen and labile carbon compounds during growth. This substrate competition may exclude dependent *Ascomycota* species, potentially limiting their proliferation. However, *Mortierellomycota* possesses a more efficient ability to decompose complex carbon sources, thus giving it a competitive advantage and increasing its relative abundance. On the other hand, the observed decrease in *Ascomycota* abundance could also be a result of competition with *Mortierellomycota*, as evidenced by their strongly opposing abundance patterns, suggesting direct or niche-based competition. Moreover, soil ECa and AMn content also play a regulatory role, as prior studies report a significant negative correlation between Ca levels and *Ascomycota* abundance [25]. However, contrasting findings exist, with some studies reporting increased *Ascomycota* abundance in morel-cultivated soils [22], and some others demonstrating site-specific variations in both *Ascomycota* and *Mortierellomycota* responses [23]. Furthermore, the presence of certain nitrogen-fixing bacteria, such as those from the *Bradyrhizobium* genus (Figure S3), could be associated with changes in the relative abundance of *Ascomycota and Mortierellomycota*. These bacteria could secrete nitrogen-containing compounds, providing a competitive advantage or exhibiting greater nitrogen requirements to *Mortierellomycota* over *Ascomycota* in morel-cultivated soils. The specific mechanisms underlying their effects still require further systematic elucidation in future controlled experiments. Nevertheless, these discrepancies likely reflect fundamental differences between soil ecosystems (e.g., forest, greenhouse, and paddy soils) [26], particularly in their different edaphic properties, including pH, organic matter, and mineral nutrient availability.

In contrast to fungal communities, bacterial communities in morel-cultivated soils did not show statistically significant differences compared to the control. Actually, both α diversity and the dominant bacterial phyla (*Proteobacteria*, *Bacteroidetes*, and *Acidobacteria*) remained consistent between cultivated and uncultivated soils. This observed bacterial community stability likely stems from their fundamental ecological roles and physiological characteristics [27]. As the primary decomposers of labile organic compounds and key drivers of biogeochemical cycles, soil bacteria possess exceptionally high metabolic plasticity and rapid adaptability to environmental changes. These advantages may render soil bacterial communities less susceptible to the disturbances associated with morels compared to fungi, explaining their minimal response to cultivation practices.

### 4.2. Effects of Morel Cultivation on Soil Mineral Elements

Morel cultivation exerted diverse effects on soil mineral elements in this study. The cultivation of different *Morchella* species led to an obvious decrease in the content of available boron (AB) while increasing the content of available manganese (AMn) and exchangeable calcium (ECa). The marked AB depletion may be attributed to its preferential uptake by morel mycelium and soil microorganisms, or high solubility leading to leaching losses from irrigation [28,29]. Conversely, ECa accumulation may originate from the mycelial secretion of oxalic acid solubilizing Ca from mineral complexes, or bound calcium from organic complexes, or the active translocation of calcium ions through hyphal networks from nutrient bags to the rhizosphere and soils [17].

The effects of morel cultivation on soil mineral elements vary across studies. For example, our results showed no significant change in available potassium (AK) content; this is contrasted with Lu et al. [24], who reported a decrease, while other studies document increased AK levels [27,30]. These inconsistences may be caused by divergent soil characteristics (e.g., pH, organic matter content, or clay composition) [23]. Furthermore, the change in soil mineral content during the morel growth cycle—ranging from mycelial colonization to fruiting body formation—may also contribute to these inconsistencies. Notably, the mineral content in *M. eximia*-cultivated soils exhibited distinct trends compared to other *Morchella* species. For instance, *M. eximia* soils had the lowest AK content and the highest ECa content among all treatments. The EMg content in the *M. eximia* soils was higher than in the control, contrasting with the other treatment groups. These unique patterns warrant further investigation to determine their implications for the mixed cultivation of different *Morchella* species.

### 4.3. Practical Implications and Future Directions

This study provides a comprehensive analysis of the interactions between morel cultivation, soil microbial communities, and mineral nutrient dynamics. Our findings demonstrate a strong correlation between yield, exchangeable calcium, and fungal communities. These results position specific morel like *Morchella eximia* could be as a valuable species for improving soil calcium availability while achieving productive yields. 

In practical production, soil nitrate nitrogen or microbial fertilizer amendments could be employed during morel field cultivation. For example, empirical evidence from industrial-scale morel cultivation indicates that nitrogen-fixing bacteria, such as *Azotobacter* and *Rhizobium*, significantly enhance yield optimization. These bacteria contribute to soil nitrogen pools and reduce nutrient competition.

Future work could establish long-term ecological monitoring to evaluate the species-specific impacts of *Morchella* cultivation on soil systems, examine fungal community resilience and *Ascomycota* recovery patterns after harvesting, and develop sustainable cultivation techniques that balance microbial conservation with yield optimization.

## 5. Conclusions

Morel cultivation exerts profound and species-specific impacts on soil ecosystems, with particularly significant effects on fungal communities and mineral nutrient levels. Our findings reveal that cultivation consistently reduced fungal α-diversity, particularly in high-yield species (e.g., *M. sextelata*), and drove phylum level shifts (depleting *Ascomycota* while enriching *Mortierellomycota*). These changes likely stem from competitive exclusion, nitrate-dependent niche competition (evidenced by selective NO_3_^−^-N depletion), and mineral elements like exchangeable Ca. In contrast, bacterial communities remained relatively stable across species, due to their metabolic flexibility and adaptability to nutrient fluctuations. Additionally, soil mineral changes were species-dependent, with exchangeable Ca emerging as a key yield correlate. The universal decline in available B (most pronounced in *M. importuna* and *M. sextelata*) and increase in exchangeable Ca (peaking in *M. eximia*) suggest mycelial translocation and species-specific nutrient mobilization strategies. These results highlight the interconnected ‘*Morchella* species–microbe–mineral’ relationships in morel cultivation systems. Future research should focus on elucidating the mechanisms underlying fungal diversity loss and exploring intercropping systems to maximize the ecological and economic benefits of morel cultivation.

## Figures and Tables

**Table 1:** Soil physiochemical characteristics of different *Morchella* species.

	Control	*M. importuna*	*M. sextelata*	*M. exuberans*	*M. eximia*	*Mel-13*	*Mel-21*
pH	7.85 ± 0.04	7.76 ± 0.05	7.75 ± 0.04	7.84 ± 0.06	7.92 ± 0.08	7.88 ± 0.1	7.77 ± 0.05
EC	279.44 ± 26.61	239.11 ± 7.93	268.00 ± 22.33	218.00 ± 15.21	269.67 ± 13.24	246.11 ± 3.07	260.56 ± 6.28
SOC	13.57 ± 0.17	11.70 ± 0.34	12.18 ± 0.31	12.18 ± 0.50	11.00 ± 1.2	12.12 ± 0.48	12.95 ± 0.38
TN	0.13 ± 0.00	0.13 ± 0.00	0.14 ± 0.00	0.14 ± 0.01	0.13 ± 0.00	0.14 ± 0.00	0.14 ± 0.01
AcP	51.78 ± 6.96	40.17 ± 5.54	48.45 ± 5.84	45.12 ± 8.37	40.39 ± 2.41	43.13 ± 4.87	46.75 ± 8.79
AK	224.51 ± 11.39	230.49 ± 4.98	234.90 ± 1.71	232.21 ± 4.91	209.71 ± 3.15	222.51 ± 5.63	224.96 ± 5.04
AFe	8.023 ± 0.38	8.97 ± 0.14	8.75 ± 0.48	10.02 ± 0.69	10.69 ± 0.47	8.33 ± 0.48	10.26 ± 1.06
AZn	1.31 ± 0.23	1.12 ± 0.26	1.70 ± 0.25	1.70 ± 0.45	1.66 ± 0.28	1.23 ± 0.41	1.66 ± 0.26
ACu	1.50 ± 0.07	1.59 ± 0.06	1.72 ± 0.05	1.88 ± 0.26	1.48 ± 0.07	1.34 ± 0.01	1.66 ± 0.14
ENa	1.81 ± 0.02	1.75 ± 0.00	1.78 ± 0.01	1.80 ± 0.02	1.78 ± 0.01	1.79 ± 0.01	1.78 ± 0.02

Notes: Values are mean values ± SE (*n* = 3). EC (us cm^−1^), electric conductivity; SOC (g kg^−1^), soil organic carbon; TN (%), total nitrogen; AcP (mg kg^−1^), available P; AK (mg kg^−1^), available K; AFe (mg kg^−1^), available Fe; AZn (mg kg^−1^), available Zn; ACu (mg kg^−1^), available Cu; ENa (cmol kg^−1^), exchangeable Na. No significance letters were assigned as results were not statistically different (*p* > 0.05).

## Data Availability

The original contributions presented in this study are included in the article. Further inquiries can be directed to the corresponding authors.

## Supplementary Materials

The following supporting information can be downloaded at: https://www.mdpi.com/article/10.3390/jof11060405/s1, Figure S1. The soil enzyme activities under different morel species treatments. Figure S2. Shannon index of bacteria (A) and fungi (B). Figure S3. The Pearson correlation among soil physicochemical characteristics, fungal phyla and the genus of nitrogen-fixing bacteria.
