# Supplementary material for: Interactions Between Morel Cultivation, Soil Microbes, and Mineral Nutrients: Impacts and Mechanisms
Source: J Fungi (Basel). 2025 May 24;11(6):405. doi: 10.3390/jof11060405 (PMC12193994; doi:10.3390/jof11060405)
Supplement: Supplementary file 1 [file jof-11-00405-s001.zip › jof-3602664-supplementary.pdf]

Journal of Fungi

Article

Interactions Between Morel Cultivation, Soil Microbes, and Mineral Nutrients: Impacts and Mechanisms

Yiwen Fu [1,2,†], Muxin Fan [1,†], Haiyan Qin [1], Zeyu Zhang [1], Shijun Liu [1], Shuwen Wu [1], Yun Wang [1] and Xia Yuan [1,*]

[1] School of Life and Environmental Sciences, Hangzhou Normal University, Hangzhou 311121, China; fuyiwen@caas.cn (Y.F.); 13133392524@163.com (M.F.); qhy10427@163.com (H.Q.); zhangzeyu33@163.com (Z.Z.); 2022210314015@stu.hznu.edu.cn (S.L.); 18258292210@163.com (S.W.); 2024112010030@stu.hznu.edu.cn (Y.W.)
[2] School of Environment and Surveying Engineering, Suzhou University, Suzhou 234000, China
* Correspondence: xyuan@hznu.edu.cn
† These authors contributed equally to this work.

Supplementary Material

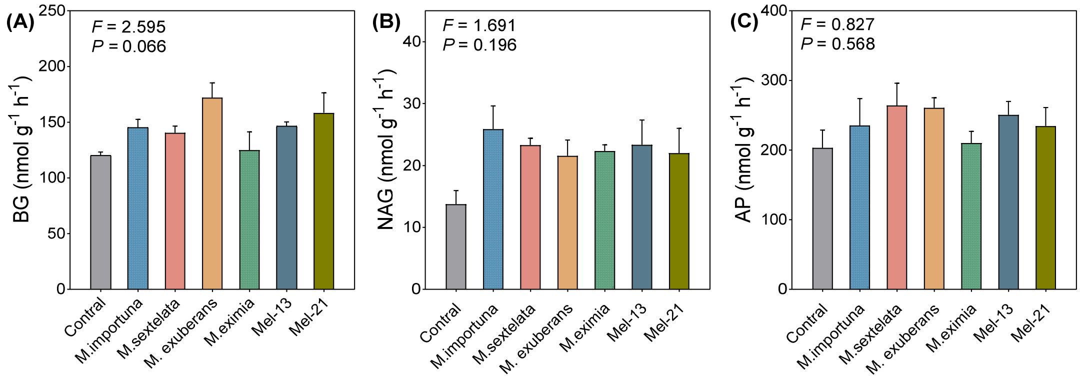

Figure S1. The soil enzyme activities under different morel species treatments. (A) BG, β–1,4–glucosidase; (B) NAG, 1, 4–N–acetyl-glucosaminidase; (C) AP, Acid phosphatase. Values are Mean ± SE (n = 3).

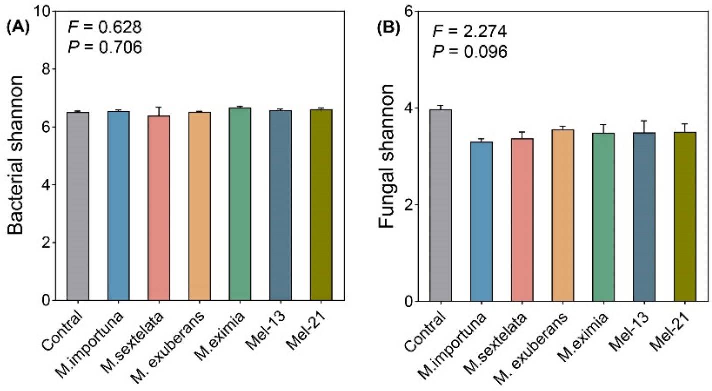

Figure S2. Shannon index of bacteria (A) and fungi (B). Values are Mean ± SE (n = 3).

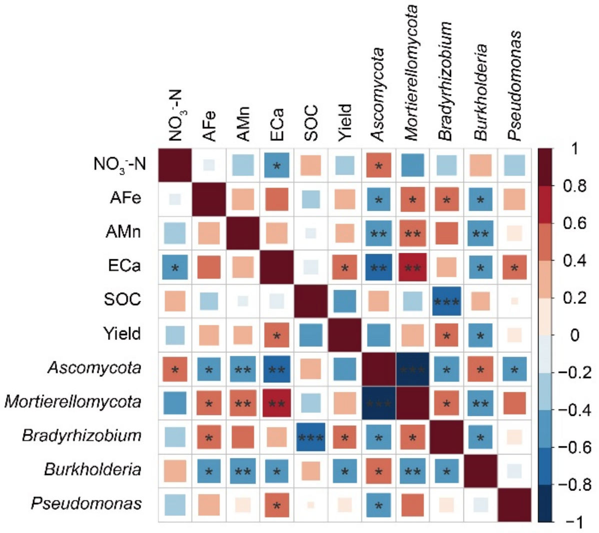

Figure S3. The Pearson correlation among soil physicochemical characteristics, fungal phyla and the genus of nitrogen-fixing bacteria. Color gradient presented Pearson's r correlation coefficient. NO_3^-–N, nitrate N; AFe, available Fe; AMn, available Mn; ECa, exchangeable Ca; SOC, soil organic carbon. * $P < 0.05$, ** $P < 0.01$, *** $P < 0.001$.
